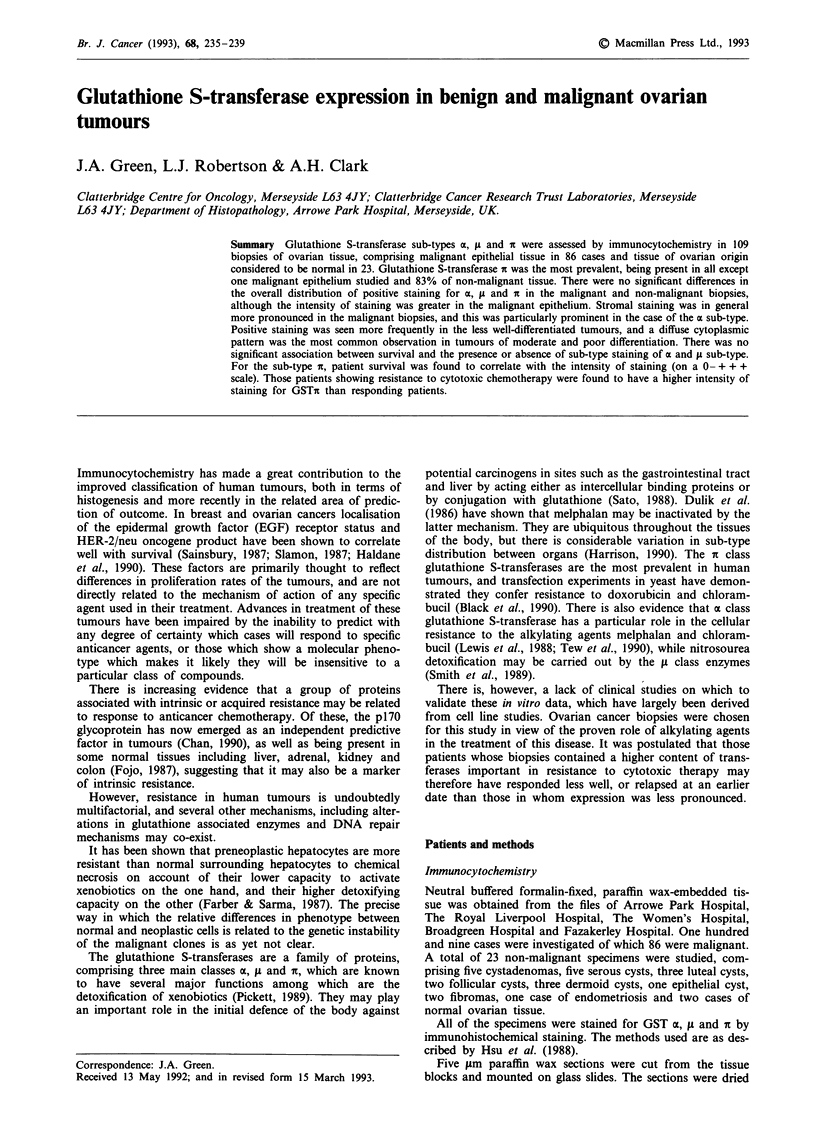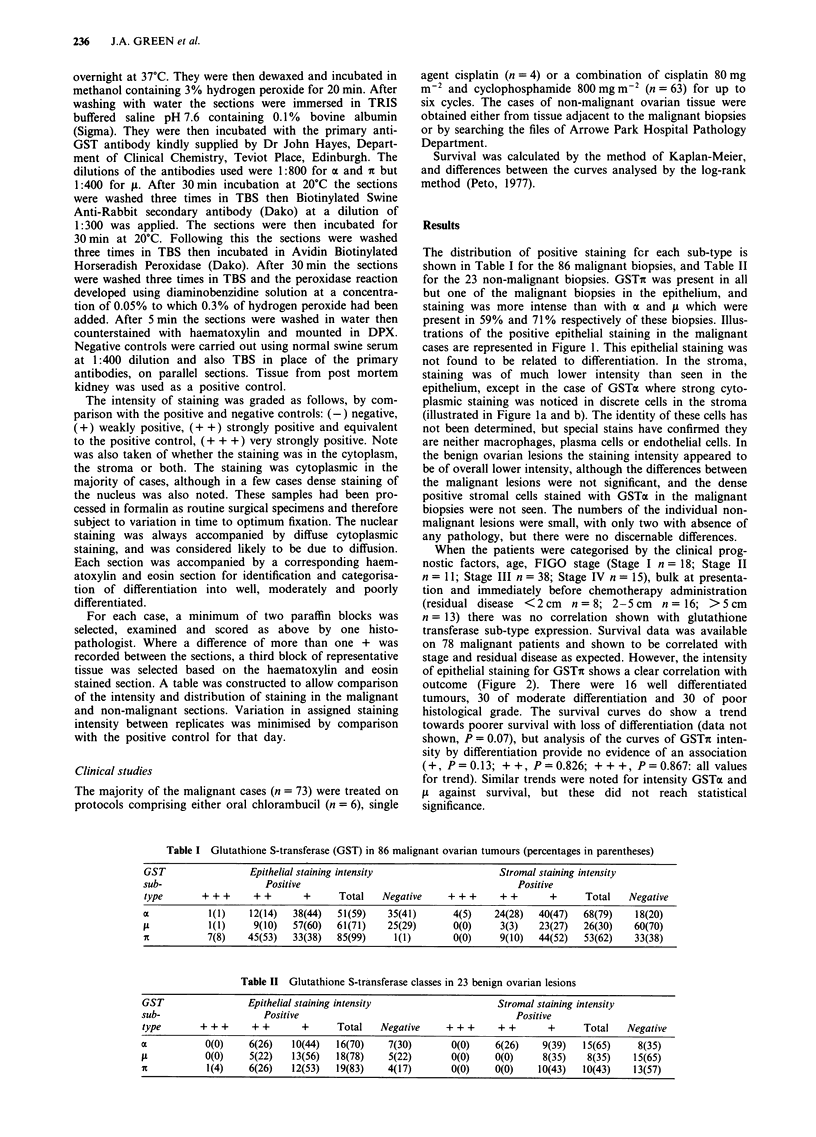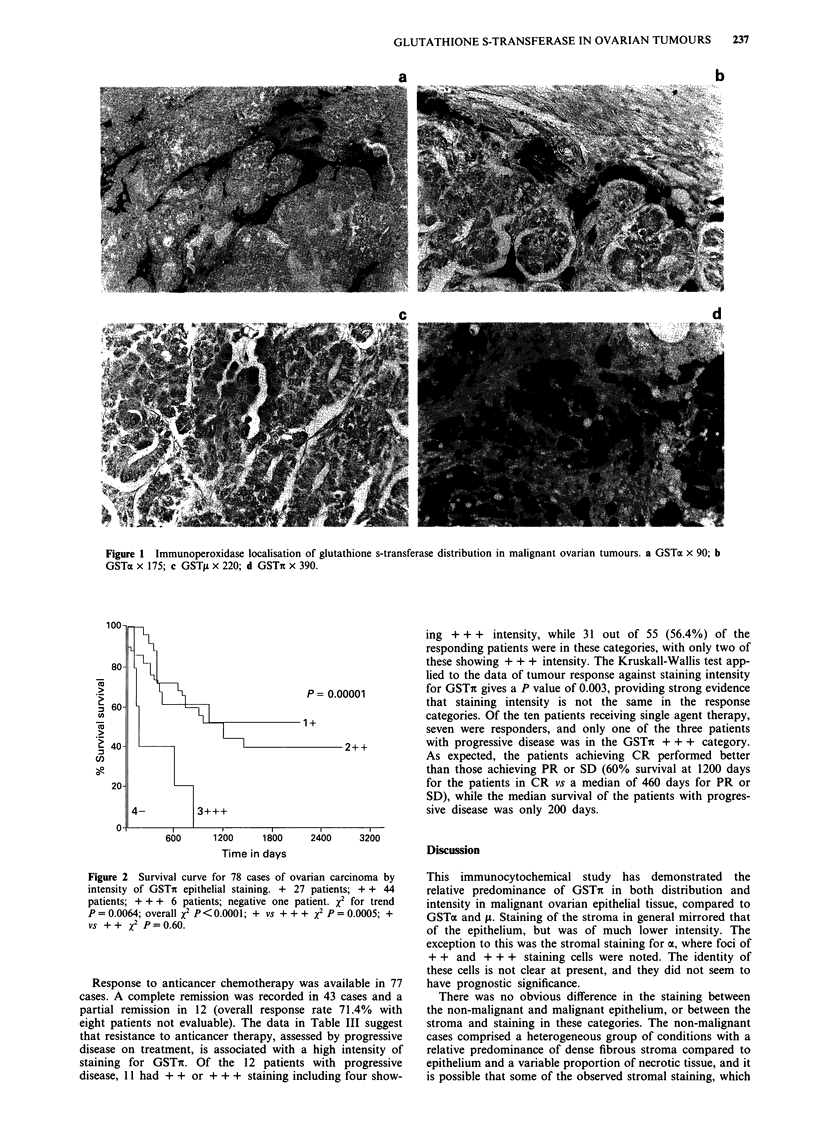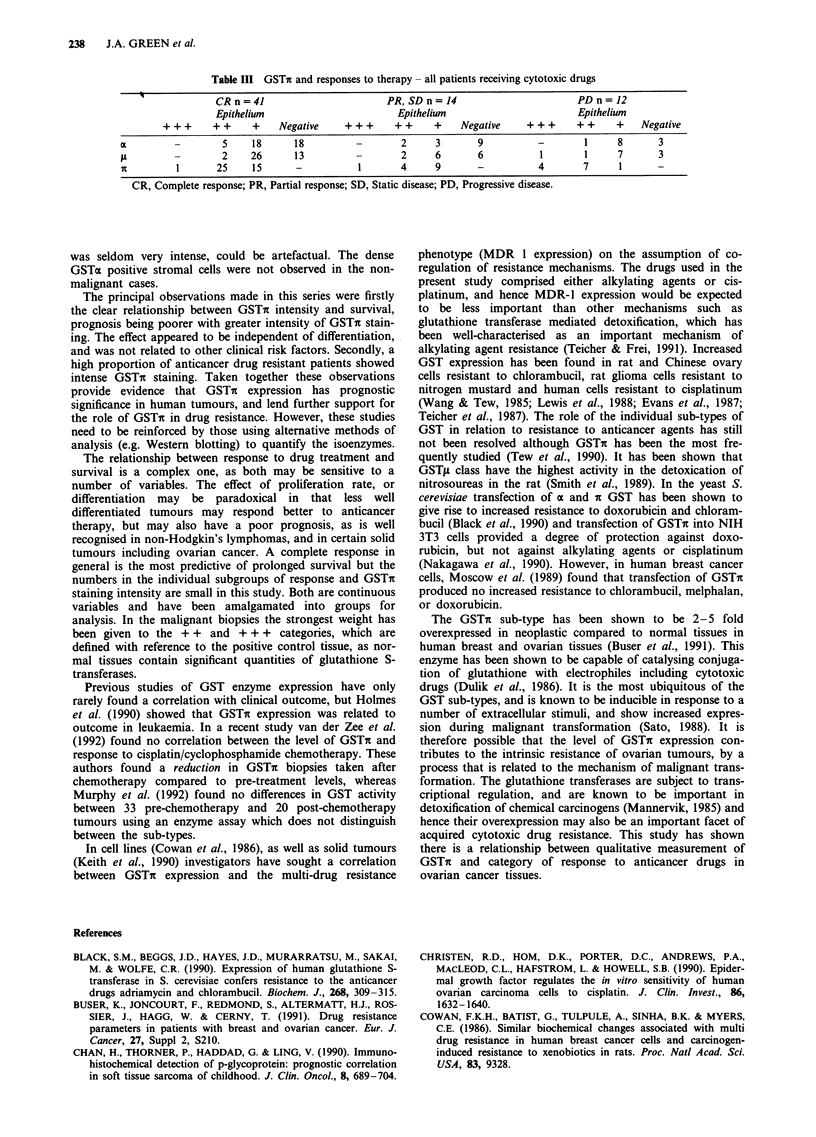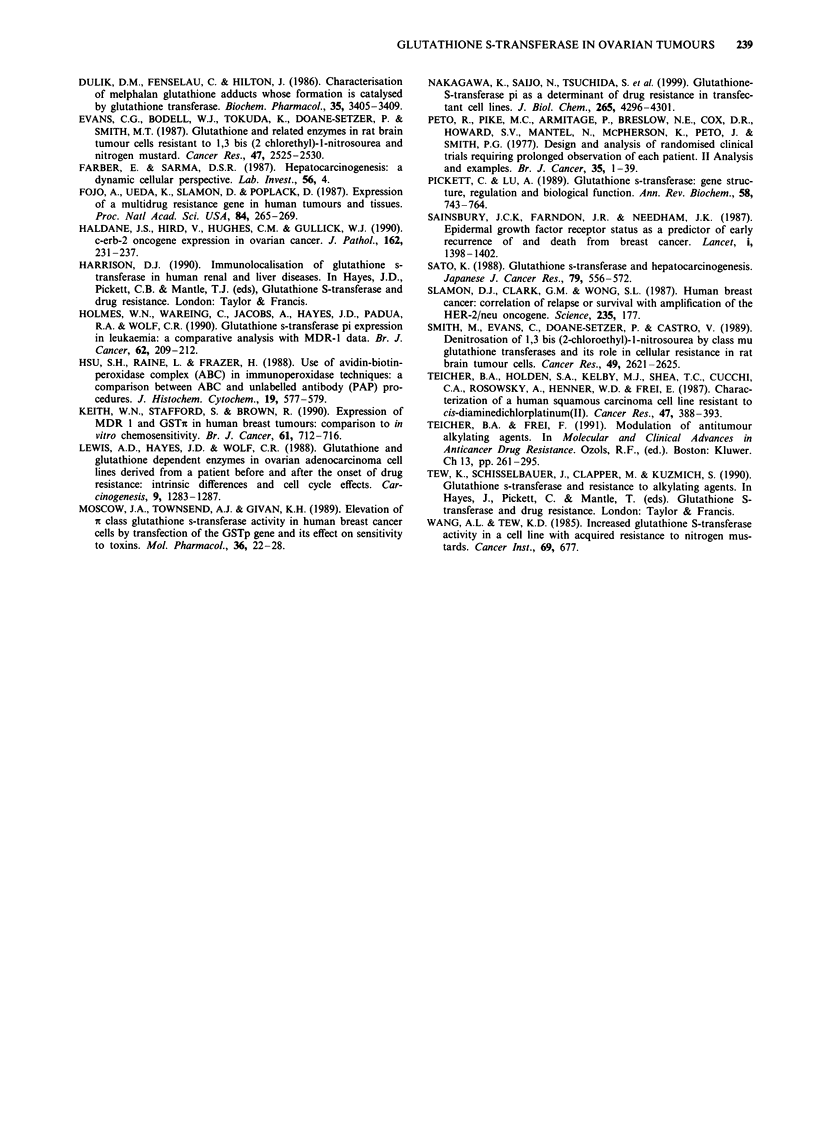# Glutathione S-transferase expression in benign and malignant ovarian tumours.

**DOI:** 10.1038/bjc.1993.321

**Published:** 1993-08

**Authors:** J. A. Green, L. J. Robertson, A. H. Clark

**Affiliations:** Clatterbridge Centre for Oncology, Merseyside, U.K.

## Abstract

**Images:**


Br. J. Cancer (1993), 68, 235-239 © Macmillan Press Ltd., 1993

### Glutathione S-transferase expression in benign and malignant ovarian tumours

J.A. Green, L.J. Robertson & A.H. Clark

*Clatterbridge Centre for Oncology, Merseyside L63 4JY; Clatterbridge Cancer Research Trust Laboratories, Merseyside L63 4JY; Department of Histopathology, Arrowe Park Hospital, Merseyside, UK.*

**Summary** Glutathione S-transferase sub-types α, μ and π were assessed by immunocytochemistry in 109 biopsies of ovarian tissue, comprising malignant epithelial tissue in 86 cases and tissue of ovarian origin considered to be normal in 23. Glutathione S-transferase π was the most prevalent, being present in all except one malignant epithelium studied and 83% of non-malignant tissue. There were no significant differences in the overall distribution of positive staining for α, μ and π in the malignant and non-malignant biopsies, although the intensity of staining was greater in the malignant epithelium. Stromal staining was in general more pronounced in the malignant biopsies, and this was particularly prominent in the case of the α sub-type. Positive staining was seen more frequently in the less well-differentiated tumours, and a diffuse cytoplasmic pattern was the most common observation in tumours of moderate and poor differentiation. There was no significant association between survival and the presence or absence of sub-type staining of α and μ sub-type. For the sub-type π, patient survival was found to correlate with the intensity of staining (on a 0-+++ scale). Those patients showing resistance to cytotoxic chemotherapy were found to have a higher intensity of staining for GSTπ than responding patients.

Immunocytochemistry has made a great contribution to the improved classification of human tumours, both in terms of histogenesis and more recently in the related area of prediction of outcome. In breast and ovarian cancers localisation of the epidermal growth factor (EGF) receptor status and HER-2/neu oncogene product have been shown to correlate well with survival (Sainsbury, 1987; Slamon, 1987; Haldane *et al.*, 1990). These factors are primarily thought to reflect differences in proliferation rates of the tumours, and are not directly related to the mechanism of action of any specific agent used in their treatment. Advances in treatment of these tumours have been impaired by the inability to predict with any degree of certainty which cases will respond to specific anticancer agents, or those which show a molecular phenotype which makes it likely they will be insensitive to a particular class of compounds.

There is increasing evidence that a group of proteins associated with intrinsic or acquired resistance may be related to response to anticancer chemotherapy. Of these, the p170 glycoprotein has now emerged as an independent predictive factor in tumours (Chan, 1990), as well as being present in some normal tissues including liver, adrenal, kidney and colon (Fojo, 1987), suggesting that it may also be a marker of intrinsic resistance.

However, resistance in human tumours is undoubtedly multifactorial, and several other mechanisms, including alterations in glutathione associated enzymes and DNA repair mechanisms may co-exist.

It has been shown that preneoplastic hepatocytes are more resistant than normal surrounding hepatocytes to chemical necrosis on account of their lower capacity to activate xenobiotics on the one hand, and their higher detoxifying capacity on the other (Farber & Sarma, 1987). The precise way in which the relative differences in phenotype between normal and neoplastic cells is related to the genetic instability of the malignant clones is as yet not clear.

The glutathione S-transferases are a family of proteins, comprising three main classes α, μ and π, which are known to have several major functions among which are the detoxification of xenobiotics (Pickett, 1989). They may play an important role in the initial defence of the body against potential carcinogens in sites such as the gastrointestinal tract and liver by acting either as intercellular binding proteins or by conjugation with glutathione (Sato, 1988). Dulik *et al.* (1986) have shown that melphalan may be inactivated by the latter mechanism. They are ubiquitous throughout the tissues of the body, but there is considerable variation in sub-type distribution between organs (Harrison, 1990). The π class glutathione S-transferases are the most prevalent in human tumours, and transfection experiments in yeast have demonstrated they confer resistance to doxorubicin and chlorambucil (Black *et al.*, 1990). There is also evidence that α class glutathione S-transferase has a particular role in the cellular resistance to the alkylating agents melphalan and chlorambucil (Lewis *et al.*, 1988; Tew *et al.*, 1990), while nitrosourea detoxification may be carried out by the μ class enzymes (Smith *et al.*, 1989).

There is, however, a lack of clinical studies on which to validate these *in vitro* data, which have largely been derived from cell line studies. Ovarian cancer biopsies were chosen for this study in view of the proven role of alkylating agents in the treatment of this disease. It was postulated that those patients whose biopsies contained a higher content of transferases important in resistance to cytotoxic therapy may therefore have responded less well, or relapsed at an earlier date than those in whom expression was less pronounced.

### Patients and methods

### *Immunocytochemistry*

Neutral buffered formalin-fixed, paraffin wax-embedded tissue was obtained from the files of Arrowe Park Hospital, The Royal Liverpool Hospital, The Women's Hospital, Broadgreen Hospital and Fazakerley Hospital. One hundred and nine cases were investigated of which 86 were malignant. A total of 23 non-malignant specimens were studied, comprising five cystadenomas, five serous cysts, three luteal cysts, two follicular cysts, three dermoid cysts, one epithelial cyst, two fibromas, one case of endometriosis and two cases of normal ovarian tissue.

All of the specimens were stained for GST α, μ and π by immunohistochemical staining. The methods used are as described by Hsu *et al.* (1988).

Five μm paraffin wax sections were cut from the tissue blocks and mounted on glass slides. The sections were dried

Correspondence: J.A. Green.
Received 13 May 1992; and in revised form 15 March 1993.

overnight at 37°C. They were then dewaxed and incubated in methanol containing 3% hydrogen peroxide for 20 min. After washing with water the sections were immersed in TRIS buffered saline pH 7.6 containing 0.1% bovine albumin (Sigma). They were then incubated with the primary anti-GST antibody kindly supplied by Dr John Hayes, Department of Clinical Chemistry, Teviot Place, Edinburgh. The dilutions of the antibodies used were 1:800 for α and π but 1:400 for μ. After 30 min incubation at 20°C the sections were washed three times in TBS then Biotinylated Swine Anti-Rabbit secondary antibody (Dako) at a dilution of 1:300 was applied. The sections were then incubated for 30 min at 20°C. Following this the sections were washed three times in TBS then incubated in Avidin Biotinylated Horseradish Peroxidase (Dako). After 30 min the sections were washed three times in TBS and the peroxidase reaction developed using diaminobenzidine solution at a concentration of 0.05% to which 0.3% of hydrogen peroxide had been added. After 5 min the sections were washed in water then counterstained with haematoxylin and mounted in DPX. Negative controls were carried out using normal swine serum at 1:400 dilution and also TBS in place of the primary antibodies, on parallel sections. Tissue from post mortem kidney was used as a positive control.

The intensity of staining was graded as follows, by comparison with the positive and negative controls: (−) negative, (+) weakly positive, (+ +) strongly positive and equivalent to the positive control, (+ + +) very strongly positive. Note was also taken of whether the staining was in the cytoplasm, the stroma or both. The staining was cytoplasmic in the majority of cases, although in a few cases dense staining of the nucleus was also noted. These samples had been processed in formalin as routine surgical specimens and therefore subject to variation in time to optimum fixation. The nuclear staining was always accompanied by diffuse cytoplasmic staining, and was considered likely to be due to diffusion. Each section was accompanied by a corresponding haematoxylin and eosin section for identification and categorisation of differentiation into well, moderately and poorly differentiated.

For each case, a minimum of two paraffin blocks was selected, examined and scored as above by one histopathologist. Where a difference of more than one + was recorded between the sections, a third block of representative tissue was selected based on the haematoxylin and eosin stained section. A table was constructed to allow comparison of the intensity and distribution of staining in the malignant and non-malignant sections. Variation in assigned staining intensity between replicates was minimised by comparison with the positive control for that day.

### *Clinical studies*

The majority of the malignant cases ($n = 73$) were treated on protocols comprising either oral chlorambucil ($n = 6$), single agent cisplatin ($n = 4$) or a combination of cisplatin 80 mg $m^{-2}$ and cyclophosphamide 800 mg $m^{-2}$ ($n = 63$) for up to six cycles. The cases of non-malignant ovarian tissue were obtained either from tissue adjacent to the malignant biopsies or by searching the files of Arrowe Park Hospital Pathology Department.

Survival was calculated by the method of Kaplan-Meier, and differences between the curves analysed by the log-rank method (Peto, 1977).

### Results

The distribution of positive staining fcr each sub-type is shown in Table I for the 86 malignant biopsies, and Table II for the 23 non-malignant biopsies. GSTπ was present in all but one of the malignant biopsies in the epithelium, and staining was more intense than with α and μ which were present in 59% and 71% respectively of these biopsies. Illustrations of the positive epithelial staining in the malignant cases are represented in Figure 1. This epithelial staining was not found to be related to differentiation. In the stroma, staining was of much lower intensity than seen in the epithelium, except in the case of GSTα where strong cytoplasmic staining was noticed in discrete cells in the stroma (illustrated in Figure 1a and b). The identity of these cells has not been determined, but special stains have confirmed they are neither macrophages, plasma cells or endothelial cells. In the benign ovarian lesions the staining intensity appeared to be of overall lower intensity, although the differences between the malignant lesions were not significant, and the dense positive stromal cells stained with GSTα in the malignant biopsies were not seen. The numbers of the individual non-malignant lesions were small, with only two with absence of any pathology, but there were no discernable differences.

When the patients were categorised by the clinical prognostic factors, age, FIGO stage (Stage I $n = 18$; Stage II $n = 11$; Stage III $n = 38$; Stage IV $n = 15$), bulk at presentation and immediately before chemotherapy administration (residual disease $<2$ cm $n = 8$; 2–5 cm $n = 16$; $>5$ cm $n = 13$) there was no correlation shown with glutathione transferase sub-type expression. Survival data was available on 78 malignant patients and shown to be correlated with stage and residual disease as expected. However, the intensity of epithelial staining for GSTπ shows a clear correlation with outcome (Figure 2). There were 16 well differentiated tumours, 30 of moderate differentiation and 30 of poor histological grade. The survival curves do show a trend towards poorer survival with loss of differentiation (data not shown, $P = 0.07$), but analysis of the curves of GSTπ intensity by differentiation provide no evidence of an association (+, $P = 0.13$; + +, $P = 0.826$; + + +, $P = 0.867$: all values for trend). Similar trends were noted for intensity GSTα and μ against survival, but these did not reach statistical significance.

**Table I** Glutathione S-transferase (GST) in 86 malignant ovarian tumours (percentages in parentheses)

| *GST sub-type* | *Epithelial staining intensity Positive* + + + | + + | + | Total | *Negative* | *Stromal staining intensity Positive* + + + | + + | + | Total | *Negative* |
|---|---|---|---|---|---|---|---|---|---|---|
| α | 1(1) | 12(14) | 38(44) | 51(59) | 35(41) | 4(5) | 24(28) | 40(47) | 68(79) | 18(20) |
| μ | 1(1) | 9(10) | 57(60) | 61(71) | 25(29) | 0(0) | 3(3) | 23(27) | 26(30) | 60(70) |
| π | 7(8) | 45(53) | 33(38) | 85(99) | 1(1) | 0(0) | 9(10) | 44(52) | 53(62) | 33(38) |

**Table II** Glutathione S-transferase classes in 23 benign ovarian lesions

| *GST sub-type* | *Epithelial staining intensity Positive* + + + | + + | + | Total | *Negative* | *Stromal staining intensity Positive* + + + | + + | + | Total | *Negative* |
|---|---|---|---|---|---|---|---|---|---|---|
| α | 0(0) | 6(26) | 10(44) | 16(70) | 7(30) | 0(0) | 6(26) | 9(39) | 15(65) | 8(35) |
| μ | 0(0) | 5(22) | 13(56) | 18(78) | 5(22) | 0(0) | 0(0) | 8(35) | 8(35) | 15(65) |
| π | 1(4) | 6(26) | 12(53) | 19(83) | 4(17) | 0(0) | 0(0) | 10(43) | 10(43) | 13(57) |

**Figure 1** Immunoperoxidase localisation of glutathione s-transferase distribution in malignant ovarian tumours. **a** GSTα × 90; **b** GSTα × 175; **c** GSTμ × 220; **d** GSTπ × 390.

**Figure 2** Survival curve for 78 cases of ovarian carcinoma by intensity of GSTπ epithelial staining. + 27 patients; ++ 44 patients; +++ 6 patients; negative one patient. $\chi^2$ for trend $P = 0.0064$; overall $\chi^2$ $P < 0.0001$; + *vs* +++ $\chi^2$ $P = 0.0005$; + *vs* ++ $\chi^2$ $P = 0.60$.

Response to anticancer chemotherapy was available in 77 cases. A complete remission was recorded in 43 cases and a partial remission in 12 (overall response rate 71.4% with eight patients not evaluable). The data in Table III suggest that resistance to anticancer therapy, assessed by progressive disease on treatment, is associated with a high intensity of staining for GSTπ. Of the 12 patients with progressive disease, 11 had ++ or +++ staining including four showing +++ intensity, while 31 out of 55 (56.4%) of the responding patients were in these categories, with only two of these showing +++ intensity. The Kruskall-Wallis test applied to the data of tumour response against staining intensity for GSTπ gives a *P* value of 0.003, providing strong evidence that staining intensity is not the same in the response categories. Of the ten patients receiving single agent therapy, seven were responders, and only one of the three patients with progressive disease was in the GSTπ +++ category. As expected, the patients achieving CR performed better than those achieving PR or SD (60% survival at 1200 days for the patients in CR *vs* a median of 460 days for PR or SD), while the median survival of the patients with progressive disease was only 200 days.

### Discussion

This immunocytochemical study has demonstrated the relative predominance of GSTπ in both distribution and intensity in malignant ovarian epithelial tissue, compared to GSTα and μ. Staining of the stroma in general mirrored that of the epithelium, but was of much lower intensity. The exception to this was the stromal staining for α, where foci of ++ and +++ staining cells were noted. The identity of these cells is not clear at present, and they did not seem to have prognostic significance.

There was no obvious difference in the staining between the non-malignant and malignant epithelium, or between the stroma and staining in these categories. The non-malignant cases comprised a heterogeneous group of conditions with a relative predominance of dense fibrous stroma compared to epithelium and a variable proportion of necrotic tissue, and it is possible that some of the observed stromal staining, which

**Table III** GSTπ and responses to therapy – all patients receiving cytotoxic drugs

| | *CR* n = *41* *Epithelium* | | | | *PR, SD* n = *14* *Epithelium* | | | | *PD* n = *12* *Epithelium* | | | |
|---|---|---|---|---|---|---|---|---|---|---|---|---|
| | + + + | + + | + | *Negative* | + + + | + + | + | *Negative* | + + + | + + | + | *Negative* |
| α | – | 5 | 18 | 18 | – | 2 | 3 | 9 | – | 1 | 8 | 3 |
| μ | – | 2 | 26 | 13 | – | 2 | 6 | 6 | 1 | 1 | 7 | 3 |
| π | 1 | 25 | 15 | – | 1 | 4 | 9 | – | 4 | 7 | 1 | – |

CR, Complete response; PR, Partial response; SD, Static disease; PD, Progressive disease.

was seldom very intense, could be artefactual. The dense GSTα positive stromal cells were not observed in the non-malignant cases.

The principal observations made in this series were firstly the clear relationship between GSTπ intensity and survival, prognosis being poorer with greater intensity of GSTπ staining. The effect appeared to be independent of differentiation, and was not related to other clinical risk factors. Secondly, a high proportion of anticancer drug resistant patients showed intense GSTπ staining. Taken together these observations provide evidence that GSTπ expression has prognostic significance in human tumours, and lend further support for the role of GSTπ in drug resistance. However, these studies need to be reinforced by those using alternative methods of analysis (e.g. Western blotting) to quantify the isoenzymes.

The relationship between response to drug treatment and survival is a complex one, as both may be sensitive to a number of variables. The effect of proliferation rate, or differentiation may be paradoxical in that less well differentiated tumours may respond better to anticancer therapy, but may also have a poor prognosis, as is well recognised in non-Hodgkin's lymphomas, and in certain solid tumours including ovarian cancer. A complete response in general is the most predictive of prolonged survival but the numbers in the individual subgroups of response and GSTπ staining intensity are small in this study. Both are continuous variables and have been amalgamated into groups for analysis. In the malignant biopsies the strongest weight has been given to the + + and + + + categories, which are defined with reference to the positive control tissue, as normal tissues contain significant quantities of glutathione S-transferases.

Previous studies of GST enzyme expression have only rarely found a correlation with clinical outcome, but Holmes *et al.* (1990) showed that GSTπ expression was related to outcome in leukaemia. In a recent study van der Zee *et al.* (1992) found no correlation between the level of GSTπ and response to cisplatin/cyclophosphamide chemotherapy. These authors found a *reduction* in GSTπ biopsies taken after chemotherapy compared to pre-treatment levels, whereas Murphy *et al.* (1992) found no differences in GST activity between 33 pre-chemotherapy and 20 post-chemotherapy tumours using an enzyme assay which does not distinguish between the sub-types.

In cell lines (Cowan *et al.*, 1986), as well as solid tumours (Keith *et al.*, 1990) investigators have sought a correlation between GSTπ expression and the multi-drug resistance phenotype (MDR 1 expression) on the assumption of co-regulation of resistance mechanisms. The drugs used in the present study comprised either alkylating agents or cisplatinum, and hence MDR-1 expression would be expected to be less important than other mechanisms such as glutathione transferase mediated detoxification, which has been well-characterised as an important mechanism of alkylating agent resistance (Teicher & Frei, 1991). Increased GST expression has been found in rat and Chinese ovary cells resistant to chlorambucil, rat glioma cells resistant to nitrogen mustard and human cells resistant to cisplatinum (Wang & Tew, 1985; Lewis *et al.*, 1988; Evans *et al.*, 1987; Teicher *et al.*, 1987). The role of the individual sub-types of GST in relation to resistance to anticancer agents has still not been resolved although GSTπ has been the most frequently studied (Tew *et al.*, 1990). It has been shown that GSTμ class have the highest activity in the detoxication of nitrosoureas in the rat (Smith *et al.*, 1989). In the yeast *S. cerevisiae* transfection of α and π GST has been shown to give rise to increased resistance to doxorubicin and chlorambucil (Black *et al.*, 1990) and transfection of GSTπ into NIH 3T3 cells provided a degree of protection against doxorubicin, but not against alkylating agents or cisplatinum (Nakagawa *et al.*, 1990). However, in human breast cancer cells, Moscow *et al.* (1989) found that transfection of GSTπ produced no increased resistance to chlorambucil, melphalan, or doxorubicin.

The GSTπ sub-type has been shown to be 2–5 fold overexpressed in neoplastic compared to normal tissues in human breast and ovarian tissues (Buser *et al.*, 1991). This enzyme has been shown to be capable of catalysing conjugation of glutathione with electrophiles including cytotoxic drugs (Dulik *et al.*, 1986). It is the most ubiquitous of the GST sub-types, and is known to be inducible in response to a number of extracellular stimuli, and show increased expression during malignant transformation (Sato, 1988). It is therefore possible that the level of GSTπ expression contributes to the intrinsic resistance of ovarian tumours, by a process that is related to the mechanism of malignant transformation. The glutathione transferases are subject to transcriptional regulation, and are known to be important in detoxification of chemical carcinogens (Mannervik, 1985) and hence their overexpression may also be an important facet of acquired cytotoxic drug resistance. This study has shown there is a relationship between qualitative measurement of GSTπ and category of response to anticancer drugs in ovarian cancer tissues.

### References

BLACK, S.M., BEGGS, J.D., HAYES, J.D., MURARRATSU, M., SAKAI, M. & WOLFE, C.R. (1990). Expression of human glutathione S-transferase in S. cerevisiae confers resistance to the anticancer drugs adriamycin and chlorambucil. *Biochem. J.*, **268**, 309–315.

BUSER, K., JONCOURT, F., REDMOND, S., ALTERMATT, H.J., ROSSIER, J., HAGG, W. & CERNY, T. (1991). Drug resistance parameters in patients with breast and ovarian cancer. *Eur. J. Cancer*, **27**, Suppl 2, S210.

CHAN, H., THORNER, P., HADDAD, G. & LING, V. (1990). Immunohistochemical detection of p-glycoprotein: prognostic correlation in soft tissue sarcoma of childhood. *J. Clin. Oncol.*, **8**, 689–704.

CHRISTEN, R.D., HOM, D.K., PORTER, D.C., ANDREWS, P.A., MACLEOD, C.L., HAFSTROM, L. & HOWELL, S.B. (1990). Epidermal growth factor regulates the *in vitro* sensitivity of human ovarian carcinoma cells to cisplatin. *J. Clin. Invest.*, **86**, 1632–1640.

COWAN, F.K.H., BATIST, G., TULPULE, A., SINHA, B.K. & MYERS, C.E. (1986). Similar biochemical changes associated with multi drug resistance in human breast cancer cells and carcinogen-induced resistance to xenobiotics in rats. *Proc. Natl Acad. Sci. USA*, **83**, 9328.

DULIK, D.M., FENSELAU, C. & HILTON, J. (1986). Characterisation of melphalan glutathione adducts whose formation is catalysed by glutathione transferase. *Biochem. Pharmacol.*, **35**, 3405–3409.

EVANS, C.G., BODELL, W.J., TOKUDA, K., DOANE-SETZER, P. & SMITH, M.T. (1987). Glutathione and related enzymes in rat brain tumour cells resistant to 1,3 bis (2 chlorethyl)-1-nitrosourea and nitrogen mustard. *Cancer Res.*, **47**, 2525–2530.

FARBER, E. & SARMA, D.S.R. (1987). Hepatocarcinogenesis: a dynamic cellular perspective. *Lab. Invest.*, **56**, 4.

FOJO, A., UEDA, K., SLAMON, D. & POPLACK, D. (1987). Expression of a multidrug resistance gene in human tumours and tissues. *Proc. Natl Acad. Sci. USA*, **84**, 265–269.

HALDANE, J.S., HIRD, V., HUGHES, C.M. & GULLICK, W.J. (1990). c-erb-2 oncogene expression in ovarian cancer. *J. Pathol.*, **162**, 231–237.

HARRISON, D.J. (1990). Immunolocalisation of glutathione s-transferase in human renal and liver diseases. In Hayes, J.D., Pickett, C.B. & Mantle, T.J. (eds), Glutathione S-transferase and drug resistance. London: Taylor & Francis.

HOLMES, W.N., WAREING, C., JACOBS, A., HAYES, J.D., PADUA, R.A. & WOLF, C.R. (1990). Glutathione s-transferase pi expression in leukaemia: a comparative analysis with MDR-1 data. *Br. J. Cancer*, **62**, 209–212.

HSU, S.H., RAINE, L. & FRAZER, H. (1988). Use of avidin-biotin-peroxidase complex (ABC) in immunoperoxidase techniques: a comparison between ABC and unlabelled antibody (PAP) procedures. *J. Histochem. Cytochem.*, **19**, 577–579.

KEITH, W.N., STAFFORD, S. & BROWN, R. (1990). Expression of MDR 1 and GSTπ in human breast tumours: comparison to *in vitro* chemosensitivity. *Br. J. Cancer*, **61**, 712–716.

LEWIS, A.D., HAYES, J.D. & WOLF, C.R. (1988). Glutathione and glutathione dependent enzymes in ovarian adenocarcinoma cell lines derived from a patient before and after the onset of drug resistance: intrinsic differences and cell cycle effects. *Carcinogenesis*, **9**, 1283–1287.

MOSCOW, J.A., TOWNSEND, A.J. & GIVAN, K.H. (1989). Elevation of π class glutathione s-transferase activity in human breast cancer cells by transfection of the GSTp gene and its effect on sensitivity to toxins. *Mol. Pharmacol.*, **36**, 22–28.

NAKAGAWA, K., SAIJO, N., TSUCHIDA, S. *et al.* (1999). Glutathione-S-transferase pi as a determinant of drug resistance in transfectant cell lines. *J. Biol. Chem.*, **265**, 4296–4301.

PETO, R., PIKE, M.C., ARMITAGE, P., BRESLOW, N.E., COX, D.R., HOWARD, S.V., MANTEL, N., MCPHERSON, K., PETO, J. & SMITH, P.G. (1977). Design and analysis of randomised clinical trials requiring prolonged observation of each patient. II Analysis and examples. *Br. J. Cancer*, **35**, 1–39.

PICKETT, C. & LU, A. (1989). Glutathione s-transferase: gene structure, regulation and biological function. *Ann. Rev. Biochem.*, **58**, 743–764.

SAINSBURY, J.C.K, FARNDON, J.R. & NEEDHAM, J.K. (1987). Epidermal growth factor receptor status as a predictor of early recurrence of and death from breast cancer. *Lancet*, **i**, 1398–1402.

SATO, K. (1988). Glutathione s-transferase and hepatocarcinogenesis. *Japanese J. Cancer Res.*, **79**, 556–572.

SLAMON, D.J., CLARK, G.M. & WONG, S.L. (1987). Human breast cancer: correlation of relapse or survival with amplification of the HER-2/neu oncogene. *Science*, **235**, 177.

SMITH, M., EVANS, C., DOANE-SETZER, P. & CASTRO, V. (1989). Denitrosation of 1,3 bis (2-chloroethyl)-1-nitrosourea by class mu glutathione transferases and its role in cellular resistance in rat brain tumour cells. *Cancer Res.*, **49**, 2621–2625.

TEICHER, B.A., HOLDEN, S.A., KELBY, M.J., SHEA, T.C., CUCCHI, C.A., ROSOWSKY, A., HENNER, W.D. & FREI, E. (1987). Characterization of a human squamous carcinoma cell line resistant to *cis*-diaminedichlorplatinum(II). *Cancer Res.*, **47**, 388–393.

TEICHER, B.A. & FREI, F. (1991). Modulation of antitumour alkylating agents. In *Molecular and Clinical Advances in Anticancer Drug Resistance*. Ozols, R.F., (ed.). Boston: Kluwer. Ch 13, pp. 261–295.

TEW, K., SCHISSELBAUER, J., CLAPPER, M. & KUZMICH, S. (1990). Glutathione s-transferase and resistance to alkylating agents. In Hayes, J., Pickett, C. & Mantle, T. (eds). Glutathione S-transferase and drug resistance. London: Taylor & Francis.

WANG, A.L. & TEW, K.D. (1985). Increased glutathione S-transferase activity in a cell line with acquired resistance to nitrogen mustards. *Cancer Inst.*, **69**, 677.